# Supplementary material for: Effects and avoidance of photoconversion-induced artifacts in confocal and STED microscopy
Source: Nat Methods. 2024 Jun 4;21(7):1171–4. doi: 10.1038/s41592-024-02297-4 (PMC11543600; doi:10.1038/s41592-024-02297-4)
Supplement: Supplementary file 1 — Reporting Summary [file 41592_2024_2297_MOESM1_ESM.pdf]

nature portfolio

Corresponding author(s): Christian Eggeling
Pablo Carravilla

Last updated by author(s): Apr 19, 2024

Reporting Summary

Nature Portfolio wishes to improve the reproducibility of the work that we publish. This form provides structure for consistency and transparency in reporting. For further information on Nature Portfolio policies, see our Editorial Policies and the Editorial Policy Checklist.

Statistics

For all statistical analyses, confirm that the following items are present in the figure legend, table legend, main text, or Methods section.

n/a	Confirmed	
☐	☒	The exact sample size (n) for each experimental group/condition, given as a discrete number and unit of measurement
☐	☒	A statement on whether measurements were taken from distinct samples or whether the same sample was measured repeatedly
☒	☐	The statistical test(s) used AND whether they are one- or two-sided<br>*Only common tests should be described solely by name; describe more complex techniques in the Methods section.*
☒	☐	A description of all covariates tested
☒	☐	A description of any assumptions or corrections, such as tests of normality and adjustment for multiple comparisons
☐	☒	A full description of the statistical parameters including central tendency (e.g. means) or other basic estimates (e.g. regression coefficient) AND variation (e.g. standard deviation) or associated estimates of uncertainty (e.g. confidence intervals)
☒	☐	For null hypothesis testing, the test statistic (e.g. F, t, r) with confidence intervals, effect sizes, degrees of freedom and P value noted<br>*Give P values as exact values whenever suitable.*
☒	☐	For Bayesian analysis, information on the choice of priors and Markov chain Monte Carlo settings
☒	☐	For hierarchical and complex designs, identification of the appropriate level for tests and full reporting of outcomes
☒	☐	Estimates of effect sizes (e.g. Cohen's d, Pearson's r), indicating how they were calculated

Our web collection on statistics for biologists contains articles on many of the points above.

Software and code

Policy information about availability of computer code

Data collection	Data were acquired using Imspector 16.3.15517 (Abberior Expert Line microscope) and STEDYCON 7.1.53 (Abberior STEDYCON microscope).
Data analysis	Fiji 1.54f was used for image analysis (https://fiji.sc). FoCuS point 1.13.156 was used for FCS curve fitting. Data analysis and plot preparation were performed in Jupyter Lab 4.1.5 using Python 3.9.16 and the following libraries: numpy 1.24.3, pandas 2.0.3, matplotlib 3.7.1 and seaborn 0.12.2.

For manuscripts utilizing custom algorithms or software that are central to the research but not yet described in published literature, software must be made available to editors and reviewers. We strongly encourage code deposition in a community repository (e.g. GitHub). See the Nature Portfolio guidelines for submitting code & software for further information.

Data

Policy information about availability of data

All manuscripts must include a data availability statement. This statement should provide the following information, where applicable:

- Accession codes, unique identifiers, or web links for publicly available datasets
- A description of any restrictions on data availability
- For clinical datasets or third party data, please ensure that the statement adheres to our policy

Data and visualisation code are available from Zenodo (https://zenodo.org/doi/10.5281/zenodo.10996577).

Human research participants

Policy information about studies involving human research participants and Sex and Gender in Research.

Reporting on sex and gender	N/A
Population characteristics	N/A
Recruitment	N/A
Ethics oversight	N/A

Note that full information on the approval of the study protocol must also be provided in the manuscript.

Field-specific reporting

Please select the one below that is the best fit for your research. If you are not sure, read the appropriate sections before making your selection.

☒ Life sciences ☐ Behavioural & social sciences ☐ Ecological, evolutionary & environmental sciences

For a reference copy of the document with all sections, see nature.com/documents/nr-reporting-summary-flat.pdf

Life sciences study design

All studies must disclose on these points even when the disclosure is negative.

Sample size	No statistical method was used for sample size determination. Three independent replicates of each experiment were performed (n=3). This sample size was resulted in low data variability (measured as the standard deviation of the independent replicates) and was considered sufficient to accurately determine the photophysical parameteres measured in this study: emission spectrum, average number of molecules, molecular brightness, average fluorescence lifetime and generalised polarisation.
Data exclusions	No data were excluded.
Replication	Three independent replicates were performed for each condition. To ensure reproducibility average values of independent experiments are considered, not individual measurements. All replication attempts were successful.
Randomization	No randomization measures. Randomization was not deemed necessary, as the results of our experiments are quantitatively measurable parameters that are not influenced by subjective bias.
Blinding	No blinding. It was not deemed necessary, as the results of our experiments are quantitatively measurable parameters that are not influenced by subjective bias.

Reporting for specific materials, systems and methods

We require information from authors about some types of materials, experimental systems and methods used in many studies. Here, indicate whether each material, system or method listed is relevant to your study. If you are not sure if a list item applies to your research, read the appropriate section before selecting a response.

Materials & experimental systems

n/a	Involved in the study	
☐	☒	Antibodies
☐	☒	Eukaryotic cell lines
☒	☐	Palaeontology and archaeology
☒	☐	Animals and other organisms
☒	☐	Clinical data
☒	☐	Dual use research of concern

Methods

n/a	Involved in the study	
☒	☐	ChIP-seq
☒	☐	Flow cytometry
☒	☐	MRI-based neuroimaging

Antibodies

Antibodies used	The following antibodies were used:

Antibodies used	Abberior STAR RED goat anti-mouse IgG (Abberior, STRED-1001), Anti-Mouse-IgG - Atto 647N antibody produced in goat (Sigma-Aldrich, 50185), Anti-Mouse IgG - Atto 655 antibody produced in goat (Sigma-Aldrich, 50283)
Validation	No validation was required as antibodies were not used for epitope recognition.

Eukaryotic cell lines

Policy information about cell lines and Sex and Gender in Research

Cell line source(s)	PtK2 (NBL-5) were acquired from ATCC (CCL-56)
Authentication	None
Mycoplasma contamination	Cells tested negative for mycoplasma.
Commonly misidentified lines (See ICLAC register)	No commonly misidentified cell lines were used in this study.
